# Hypoglossal-Facial Nerve Reconstruction Using a Y-Tube-Conduit Reduces Aberrant Synkinetic Movements of the Orbicularis Oculi and Vibrissal Muscles in Rats

**DOI:** 10.1155/2014/543020

**Published:** 2014-12-09

**Authors:** Yasemin Kaya, Umut Ozsoy, Murat Turhan, Doychin N. Angelov, Levent Sarikcioglu

**Affiliations:** ^1^Department of Anatomy, Akdeniz University Faculty of Medicine, 07070 Antalya, Turkey; ^2^Department of Ear Nose Throat, Akdeniz University Faculty of Medicine, 07070 Antalya, Turkey; ^3^Anatomical Institute I, University of Cologne, 50931 Cologne, Germany

## Abstract

The facial nerve is the most frequently damaged nerve in head and neck trauma. Patients undergoing facial nerve reconstruction often complain about disturbing abnormal synkinetic movements of the facial muscles (mass movements, synkinesis) which are thought to result from misguided collateral branching of regenerating motor axons and reinnervation of inappropriate muscles. Here, we examined whether use of an aorta Y-tube conduit during reconstructive surgery after facial nerve injury reduces synkinesis of orbicularis oris (blink reflex) and vibrissal (whisking) musculature. The abdominal aorta plus its bifurcation was harvested (*N* = 12) for Y-tube conduits. Animal groups comprised intact animals (Group 1), those receiving hypoglossal-facial nerve end-to-end coaptation alone (HFA; Group 2), and those receiving hypoglossal-facial nerve reconstruction using a Y-tube (HFA-Y-tube, Group 3). Videotape motion analysis at 4 months showed that HFA-Y-tube group showed a reduced synkinesis of eyelid and whisker movements compared to HFA alone.

## 1. Introduction

The facial nerve is the most frequently damaged nerve due to a variety of reasons including radical parotidectomy, petrous bone surgery, removal of the cerebellopontine angle tumors, and iatrogenic injury [1–5]. The resulting facial paralysis and loss of mimetic movement is a social handicap that often leads to severe psychological and economic hardship [6].

The most common surgical approach for repairing transected nerves has been direct suture of the two stumps (end-to-end anastomosis) [7] but functional recovery is poor [8]. Indeed, despite significant advances in microsurgical and neurootological techniques, functional recovery remains less than optimal [4, 8]. Furthermore, facial palsy is complete in up to 10% of patients undergoing removal of tumors located in the cerebellopontine angle [2, 4, 5, 8–10].

In cases where large segments of the intracranial or intratemporal portions of the facial nerve are destroyed, hypoglossal-facial anastomosis (HFA) is a favoured surgical technique aimed at encouraging some innervation (hypoglossal) of the facial muscles and minimizing facial palsy [4, 11, 12]. Indeed, in animal studies, it has been shown that end-to-end anastomosis of the proximal stump of a transected intact hypoglossal nerve to the distal stump of a transected facial nerve enables outgrowth of hypoglossal axons into the paralyzed facial muscles [10, 13, 14]. However, many patients receiving HFA experience disturbing abnormal synchronous movements (synkinesis) of the facial (e.g., mimic and eyelid) muscles attributable to excessive collateral axonal branching at the lesion site [15, 16] and subsequent reinnervation of inappropriate (often antagonist) muscles [4, 17–20].

We have previously shown that, following facial nerve injury and end-to-end anastomosis, collateral branching can be reduced by local application of neutralizing antibodies to neurotrophic factors [21] or by stabilizing microtubule synthesis with taxol [22]. However, neither treatment improved recovery of facial motor (whisking) function [23]. Similarly, surgical reconstruction using a Y-tube conduit to direct regeneration of the proximal stump of the hypoglossal nerve into the distal stump of the facial nerve and avoid tightly suturing (anastomosis) the nerve stumps [24] reduced axonal sprouting but also did not improve recovery of whisking [25].

In human patients receiving facial nerve reconstruction surgery, the most common synkinesis is eyelid closure during a smile. However, little is known about the effects of different types of surgical reconstruction on the likelihood of synkinesis after nerve injury.

Rats provide an ideal model to examine synkinesis because they display spontaneous bursts of activity of the vibrissal muscles (whisking) that can be analyzed in relation to eyelid closure (blink) [9, 23, 26, 27]. In intact rats, explorative whisking is not accompanied by synkinetic eyelid closure. Likewise, an “air puff” to the cornea and periorbital region triggers an eyelid closure (blink reflex) but no associated whisking of the vibrissal hairs. During facial nerve regeneration, however, excessive collateral branching of axons causes misrouting leading to aberrant reinnervation of musculature and pathological synchronized movement (synkinesis) of the orbicularis oculi [9] and vibrissal muscles [23].

We therefore examined the extent of synkinesis after facial nerve reconstruction using HFA alone or HFA using an aorta Y-tube conduit. We undertook two quantitative motion analyses: one on eyelid closure during spontaneous whisking and the other on vibrissal whisking during a blink reflex elicited by an air puff to the cornea and periorbital region. Video recordings taken at the time of data collection for a previously published experiment [25] were used for analysis.

## 2. Material and Methods

### 2.1. Animals

The data were collected, but not previously reported, from animals used in an earlier study [25]. Forty-eight young adult female inbred rats (origin Harlan Laboratories Israel, strain “Wistar,” F11 generation) were obtained from the Laboratory Animal Unit of the Akdeniz University. Twelve animals were used for harvesting their abdominal aorta plus its bifurcation. The remainder were split into 3 groups (*n* = 12 in each) comprising intact controls (Group 1), rats subjected to hypoglossal-facial nerve end-to-end coaptation alone (HFA; Group 2), and those receiving hypoglossal-facial nerve reconstruction using a Y-tube (HFA-Y-tube, Group 3). After 4 months, all animals were subjected to video-based motion analysis of orbicularis oculi and vibrissal motor performance.

Throughout, allocation was concealed; that is, the person undertaking the surgery did not know to which group the animal would be allocated. Animals were randomised into groups using a randomised number sequence and assessment was blinded.

### 2.2. Surgical Procedures

#### 2.2.1. Preparation of Y-Tube

The aortal bifurcation into the right and left common iliac arteries was used as a Y-tube conduit. In brief, once exposed, an artery clamp was applied to the aorta just below the diaphragm to avoid excessive bleeding. The aorta was cut and traced inferiorly taking care to avoid punctures and a 1.5–2.0 cm long segment including the common iliac arteries removed and irrigated with phosphate buffer to remove blood clots. Small branches originating from the aorta were cauterized.

#### 2.2.2. Nerve Reconstruction

Animals were anesthetized with a mixture of ketamine (100 mg/kg, i.p.) and xylazine HCl (15 mg/kg; i.p.). Hypoglossal-facial anastomosis (HFA; Group 2) was performed unilaterally (right side) as described previously [10]. In brief, after exposure under an operating microscope with fiberoptic illumination (Olympus SZ61), the glandula lacrimalis extraorbitalis was retracted laterally to approach the proximal part of the buccal and zygomatic branches. The facial vein crossing over the zygomatic and buccal branches was cauterized and the zygomatic and buccal branches were cut at the level of their origin from main trunk of the facial nerve. The remaining part of the facial nerve trunk was ligated with 9.0 silk suture. The hypoglossal nerve was approached by retraction of the posterior belly of the digastric and stylohyoid muscles. The hypoglossal nerve was cut just before its division into medial and lateral branches. The proximal end of the hypoglossal nerve was directly apposed (i.e., no interstump distance) to the distal stump of the facial nerve and sutured with two 9.0 sutures.

**Figure 1:**
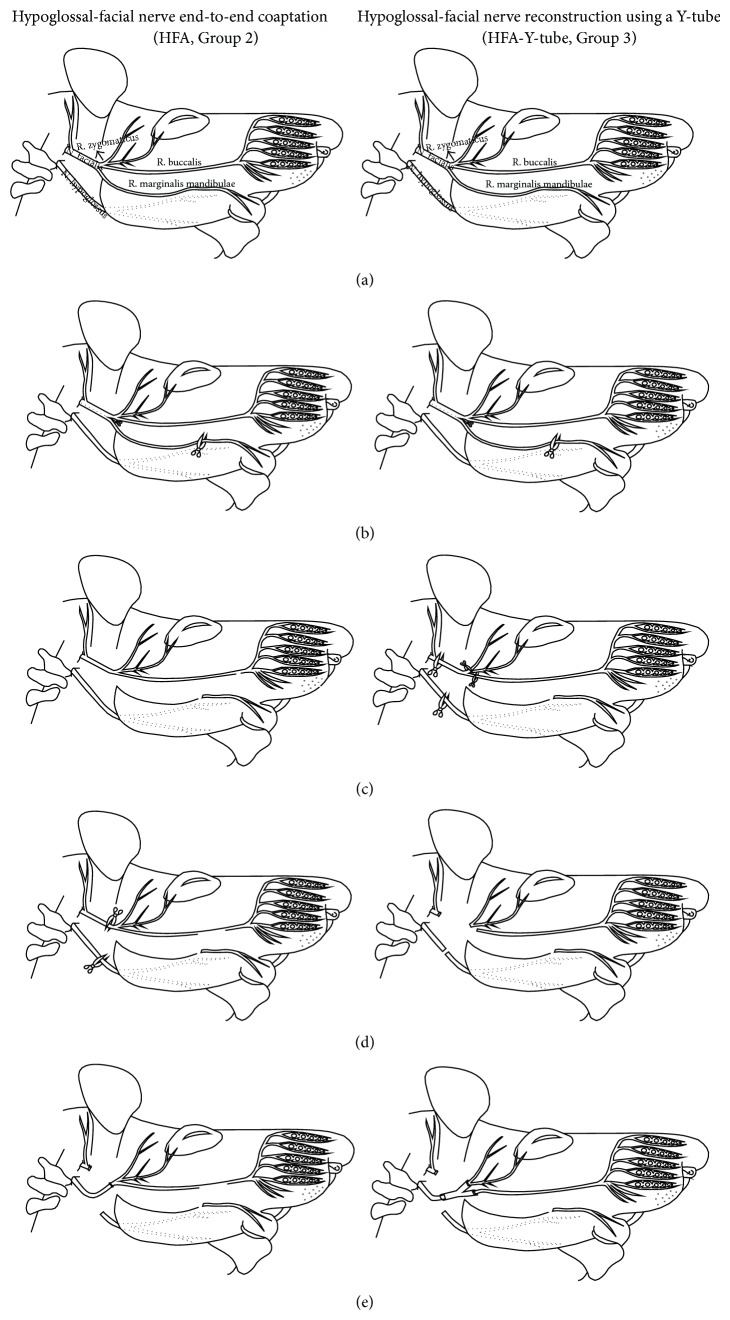
Drawing of the surgical procedures in Groups 2 and 3. (a) Normal anatomy of the facial and hypoglossal nerves, (b)–(d) steps of the nerve reconstruction. (e) Final step for hypoglossal-facial nerve reconstruction by using Aorta-Y-tube conduit or classical coaptation.

HFA using a Y-tube has also been described previously [10, 25]. Procedures were as above except that, to avoid any delay to implantation, Y-tube preparation from one set of animals and exposure of nerve branches from the other set were accomplished simultaneously by 2 separate surgical teams. In addition, alignment of the proximal hypoglossal nerve stump and the distal facial nerve stump within the Y-tube involved an interstump distance of 0.5 cm. The Y-tube was shortened to match the interstump distance of 0.5 cm. The proximal stump of hypoglossal nerve was entubulated into the long branch of the aorta-Y-tube conduit. The distal stumps of the zygomatic and buccal nerve branches were then entubulated into the short branches of the aorta-Y-tube conduit. Two 10.0 sutures were used to secure each stump to the Y-tube (Figure 1).

### 2.3. Natural Whisker Movements

Rodents make a number of different movements with their whiskers, namely, (i) large-amplitude “explorative” sweeps of the vibrissae (in the frequency range of 5–11 Hz); (ii) low-amplitude “foveal” or “palpating” whisker movements at 15–25 Hz [28, 29]; and (iii) denervation-induced tremor that occurs after facial nerve transection [30]. All whisker movements are elicited by contractions of the intrinsic and extrinsic vibrissal musculature which are controlled solely by the facial nerve [31]. In this study, we analyzed only the large amplitude exploratory sweeps. Following facial nerve transection, such exploratory movements are completely abolished. However, with time, and as we show here, there is a gradual progression to varying levels of motor performance which can be readily assessed by video-based motion analysis. The technique is an entirely noninvasive approach to monitor recovery of function after facial nerve repair and avoids the use of invasive electromyography.

### 2.4. Simultaneous Monitoring of Eyelid and Whisker Movements

Video-based motion analysis of vibrissal motor performance and eyelid closure has been previously established and examined in a series of recent experiments [9, 10, 32, 33]. However, these studies did not examine the degree of synkinesis between whisker and eyelid movements.

Four months after nerve reconstruction, all animals were videotaped for 3–5 min during active whisker exploration using a digital video camera (Sony Handycam DCR-SR70 HDD Camcorder). Care was taken to handle the animals gently to avoid stress on the animal. For calibration, a ruler laid 20 cm below the recorder (i.e., at a constant angle of 180°) was videotaped before each trial. Selected sequences containing the most prominent whisks (vibrissal bouts) on the intact side contralateral to surgery were captured by a 2D/Manual Advanced Video System (WinAnalyze, Mikromak Service, Berlin, Germany). Frame rate of the video analysis was 25 frame/second. Eyelid closures were assessed from the same videotape sequences.

**Figure 2:**
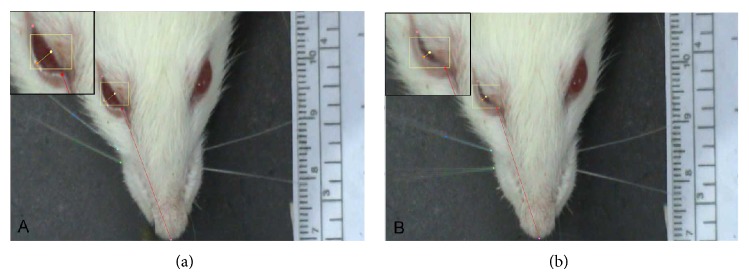
Consecutive frames of a video material showing synkinetic movement of the eyelid during whisking. Nerve reconstruction was performed on the right side of the animal. (a) Position of the vibrissal hairs and eyelid at rest. (b) Cocontraction of the orbicularis oculi muscle during exploratory whisking (protraction) of the vibrissal hairs.

To assess whisker movements, we used a geometrical model consisting of three reference points: (i) a point in the medial sagittal line close to the end of the nose, (ii) a point corresponding to the medial angle of the left orbita, and (iii) a point corresponding to the medial angle of the right orbita. Using this model we collected and evaluated data on the (i) whisking frequency (cycles of protraction and retraction per second), (ii) angle at maximal protraction (the rostrally open angle between the midsagittal plane and the hair shaft in degrees), and (iii) amplitude (the difference between maximal retraction and maximal protraction in degrees) (Figures 2(a) and 2(b)). The maximal retraction angle (in degrees) was subtracted from the maximal protraction angle and divided by the mean whisking amplitude obtained from intact animals. Values from intact animals were used as the denominator because animals undergoing facial nerve reconstruction have impaired function. This value was used to estimate whisker movements in comparison to intact animals. Normal whisking amplitude was considered to be 100%.

**Figure 3:**
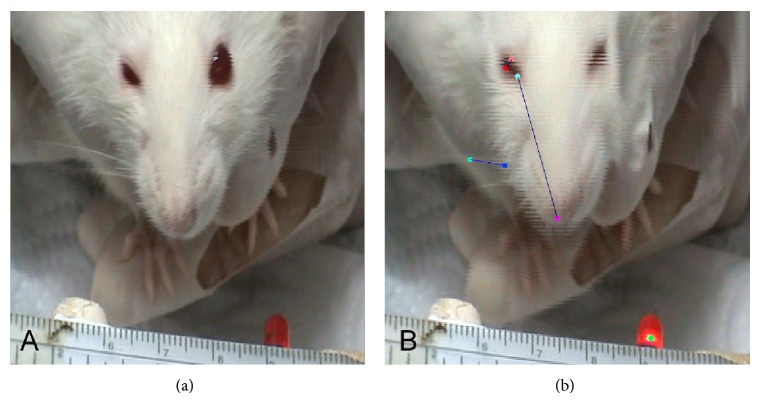
Consecutive frames of a video material showing eye closure stimulated by a “puff” stimulation and whisking at the same time. Nerve reconstruction was performed on the right side of the animal. (a) Position of the eyelids before starting the air puff. (b) Cocontraction of the orbicularis oculi muscle and vibrissal muscle during air puff stimulation.

**Figure 4:**
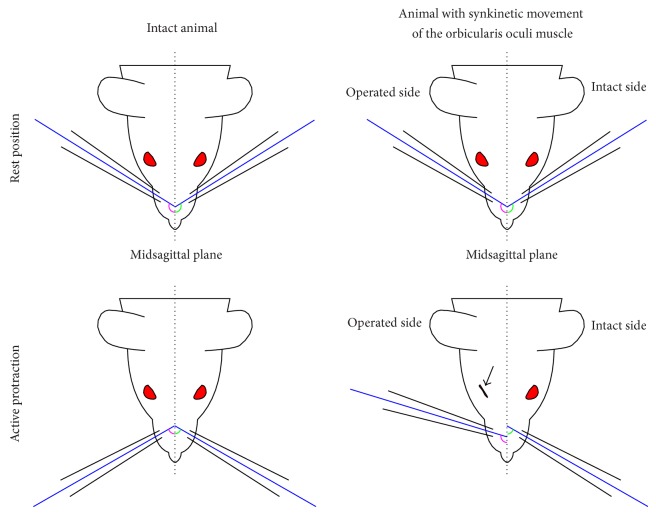
Schematic drawing of an animal showing 100% synkinetic movement of the eyelid during whisking. Arrow shows a full blink (minimal intereyelid distance) during protraction of the vibrissal hairs.

To assess spontaneous eyelid movements, video sequences were inspected on screen. In addition to the reference points used for evaluating whisking, we also added a reference point half-way along the rim of each eyelid to measure the mean distance between both eyelids. Frames were selected when eyelid closure on the intact left side was maximal, that is, when the distance between the eyelids was smallest (Figure 3). The minimal distance between the upper and lower eyelids (“inter-eyelid distance”) during a blink was subtracted from the intereyelid distance at rest and divided by the intereyelid distance at rest (formula in (1)). A full blink during whisking was considered to be 100% synkinesis of the orbicularis oculi muscle (Figure 4). Since eyelid closure in intact animals is complete (100%), any increase in the intereyelid distance indicates functional impairment.

The following are formulas used to calculate synkinetic movements:

(1) ratio  of  synchronous  movement  of  the  eyelid  during  whisking=intereyelid  distance  in  mm  in  rest  position−minimal  intereyelid  distance  in  mmwhile  maximal  protraction  of  vibrissal  hairsintereyelid  distance  in  mm  in  rest  position ×100,ratio  of  synchronous  movement  of  the  vibrissal  hair  during  blink  reflex=maximal  retraction  angle  in  degree  in  rest  position−maximal  protraction  angle  in  degreewhile  minimal  intereyelid  distancemean  whisking  amplitude  in  degree  in  intact  animals ×100.

To assess elicited eyelid movements, we used a custom-designed apparatus that delivered a single standardized volume of 20 mL air as a “puff” to the cornea and periorbital region bilaterally at a distance of 3 cm. Ten puffs were delivered sequentially over 120 s. Eyelid closure was evaluated as above.

All frames of our video material were evaluated to find the highest amplitude of the vibrissal hairs during blink reflex (contraction of the orbicularis oculi muscle) or vice versa. This is why we just concentrate to find spatial relation between whisker movement and eyelid contraction on the same sequence of the movements (the full length of the movement could be observed in 3–10 consecutive frames). Full length of the video material was examined and intereyelid distance and amplitude (in degree) of the vibrissal hair movement were measured in all sequences of the movements. For instance, when an animal contracted its orbicularis oculi muscle and showed a minimal intereyelid distance, we also measured the angular changes of the vibrissal hair to find the amplitude (we measured the retraction angle of the vibrissal hair before starting the movement and highest protraction angle while minimal intereyelid distance was observed).

It should be noted that there was a negative correlation in calculation paradigms of synkinetic movements. Minimal intereyelid distance (in mm) means more synkinetic movement. In other words during whisking if the animals show full close of its eyelid, we consider that the animal showed 100% synkinesis. However maximal protraction angle value (i.e., whisking amplitude) means more synkinetic movement.

Movements of eyelid and vibrissal hairs are reverse in direction. Function of the eyelid is to diminish palpebral rima, but function of the vibrissal muscles is to move the hairs in forward direction, for example, enlarge the amplitude. In intact animals smaller intereyelid distance will mean good function (this means that orbicularis oculi muscle contracted to decrease intereyelid distance), but larger amplitude of the vibrissal hairs will mean good function (this means that vibrissal muscle contracted and moved the vibrissal hairs in forward direction). In other words if some axons in the buccal branch showed collateral axonal branching and reinnervated both of the whisker pad and orbicularis oculi muscles, smaller intereyelid distance measured during whisking will mean more synkinesis (Figure 4).

*Data Analysis.* Data were presented as mean ± standard deviation and analyzed by ANOVA (*post hoc* Tukey test). The level of significance was set to *P* < 0.05. For analysis, GraphPad Prism version 5.0 (GraphPad Software, Inc, San Diego, Calf) was used.

## 3. Results

### 3.1. Less Synkinetic Movements of the Eyelid during Whisking in the HFA-Y-Tube Group

The formulas which were used to calculate synkinetic movements of the eyelid and vibrissal muscles are shown in (1).

**Figure 5:**
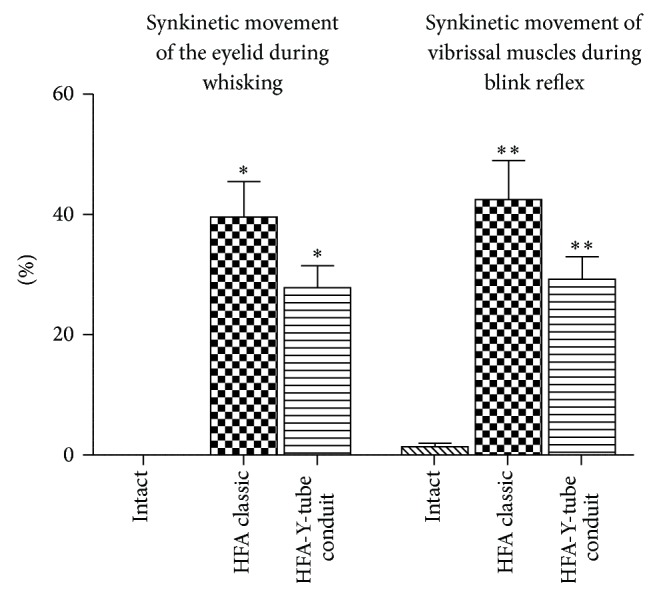
Synkinetic movement of the eyelid during whisking and synkinetic movement of the vibrissal muscles during blink reflex in two hypoglossal-facial nerve reconstruction techniques.

Synkinetic movements of the eyelid during whisking were analyzed in the frame of first experimental set. The ratio (%) of synkinetic eyelid closure occurred at the same time as whisking movement was significantly lower in the HFA+Y-tube group (27.8 ± 3.6%, mean ± SD) compared to the HFA group (39.6 ± 5.8%, *P* < 0.05, Figure 5).

### 3.2. Less Synkinetic Movement of the Vibrissal Hairs during Eye-Closure in the HFA-Y-Tube Group

Synkinetic movements of the vibrissal muscles during blink reflex were analyzed in the frame of second experimental set.

Synkinetic movement ratios were 42.4 ± 6.5% and 29.2 ± 3.7% in HFA-coaptation and HFA-Y-tube groups, respectively. There was a significant difference between both groups (Figure 5). Synkinetic movement ratios were significantly lower (*P* < 0.05) in the HFA-Y-tube conduit group compared to those of HFA-coaptation group.

## 4. Discussion

In this study, we examined the function of vibrissal and orbicularis oculi muscles after two types of hypoglossal-facial reconstruction using an objective and quantitative analysis of simultaneous movements of both muscle groups, that is, synkinesis. We showed that synkinesis was reduced following hypoglossal-facial nerve reconstruction (HFA) using a Y-tube compared to HFA-coaptation, that is, direct anastomosis, alone. Evaluation of synkinesis of eyelid and vibrissal movements may be a useful model with clinical relevance for assessing functional recovery after facial nerve injury.

### 4.1. Technical Issues

Direct measurement of orbicularis oculi electromyography in conjunction with search coil tracking in a magnetic field has been used previously as an indicator of eyelid motor activation and lid movements, respectively [34–36]. However, these techniques are invasive and have the disadvantage of potential morbidity from hardware implantation and chronic maintenance in soft tissues as well as challenges in relating EMG signals to palpebral fissure (upper and lower eyelid position). Our choice was therefore to analyze muscle function by noninvasive quantitative and objective video-based motion analysis, a well-established technique in our hands, for example, [10, 25, 27], as an indirect measure of orbicularis oculi and vibrissal muscle function.

A critical feature of our video-based assessment is to measure the amplitude of natural vibrissal bouts during free exploration. Earlier studies have shown that, during free exploration, rats rhythmically move their heads so as to bring microvibrissae into contact with the surfaces they are exploring. These periodic “microvibrissal placements” are synchronized with macrovibrissal movements not only during free exploration, but also during texture discrimination tasks [37, 38]. As a result, during our 3–5-minute video sessions, natural head movements result in some variability in whisking effort [39].

However, if the head is immobilized, the finely-tuned physiological synchrony of micro- and macrovibrissal whisking is impaired thereby introducing a confounder. Thus, in an attempt to compensate for the resulting decrease in sensory stimuli, rats react with significantly larger than normal, and therefore nonphysiological, vibrissal excursions of the order of 90 degrees [40–43].

### 4.2. Possible Role of the Retractor Bulbi Muscle on Blink Reflex

Immediately after facial nerve lesion, the retractor bulbi extraocular musculature has been shown to support blink response in rabbits [44] as well as in cats [35, 45] with its increasing activity being thought to compensate for lost orbicularis oculi function. Extraocular muscles are innervated by axons originating in the accessory abducens nucleus [45–47], not the facial nerve, and are thus spared during facial nerve lesion. Similar retractor bulbi muscles have been described in rats [47, 48], although their role in supporting blinking behavior has not been described. Further experiments will be required to assess the possible role of the retractor bulbi muscles in our model.

### 4.3. Evaluation of Synkinesis after Peripheral Nerve Injury

Synkinesis following nerve injury refers to involuntary contraction of some muscles during voluntary movement of others, for example, involuntary eye closure at the same time as smiling. This phenomenon is thought to arise from axonal misrouting during regeneration of the facial nerve [49]. Treatment options include physical therapy [50, 51], botulinum toxin injections [52], and surgical reconstruction [53, 54]. However, these have limited success and resulting peripheral facial paralysis has profound psychologic and social impacts on patients [55–57].

Improving clinical outcome requires reliable and reproducible experimental models of peripheral nerve injuries and their reconstruction [55, 58, 59]. In addition, appropriate outcome measures are also important. Various morphologic, electrophysiologic, biochemical, and functional methods have been used to assess peripheral nerve regeneration [60, 61]. However, results are variable making interpretation difficult [55, 57, 58, 61]. Although functional methods appear to relate to the success of neural regeneration, it has been difficult to establish objective and reproducible functional measures [60].

Another important issue is that we could not understand whether recovery from facial nerve cut causes abnormal whisker movements desynchronization of whisker movement, more side to side asymmetry. Although we tried to measure abnormal coactivation (contraction) of muscles, it is possible that there may be abnormal whisker synchronization. Cortical motor neurons can drive whisker movement but only of a few whiskers [62, 63], and facial motor neurons have various connections to muscles [62, 64], so it is likely the reinnervation would produce abnormal synchrony of whisker movement, not just synchronous contractions that do not normally occur. Therefore we think the further studies should be done to understand this important issue of the nerve recovery.

#### 4.3.1. Grading Systems for Evaluation of Synchronous Movements

Facial nerve palsy can be scored clinically using the Sunnybrook, House-Brackmann, and Yanagihara grading systems whose reliability has also been established [65]. Scoring systems have also been developed for animal models. Synchronous movements (0 = none; 3 = normal) of the upper lip and eyelids have been assessed in rabbits after facial nerve injury and encompass spontaneous movements as well as those elicited by light touch of the forehead and nose [66]. Another scoring system has also been developed in rats following facial nerve injury (0 = no movement; 100% = full movement) [67]. However, both systems involve stimulating the eyes and vibrissae together and are subjective.

A different scoring system [68] has been developed to assess vibrissal position and eyelid closure separately but still involves a subjective ordinal scale (absence of eyelid closure = 1; presence of orbicular muscle contraction, without blinking reflex = 2; 50% eyelid closure through blinking reflex = 3; 75% = 4; 100% eyelid closure and blinking reflex = 5. Absence of movement and posterior position of the vibrissae = 1; slight shivering and posterior position = 2; greater shivering and posterior position = 3; normal movement with posterior position = 4; symmetrical movement of the vibrissae, with anterior position = 5).

In a recent study, Hadlock et al. [69] described a method to establish normative parameters for both spontaneous and induced whisking and blinking behavior after facial nerve manipulation. Before recording the whisking and blinking behavior, they used a titanium head fixation device secured to the animal's cranium with titanium screws, with each of its four posts emerging through a separate stab incision in the skin. So, the animals were in a very stressful situation (in head-fixed position). From those animals, they continuously recorded right and left C-1 whisker positions of the animals for 5-minute sessions and continuously recorded changes in infrared detection corresponding to eye closure. They elicited whisking and blinking by delivery of olfactory stimuli (10 s scented airflows) and corneal air puffs. They reported that air puff elicited an ipsilateral blink 99% of the time, a contralateral blink 18% of the time, and changes in, or initiation of, bilateral whisking 70% of the time. Olfactory stimulus delivery prompted a change in whisking behavior 83% of the time and eye closure 20% of the time. In agreement with this study, we consider that the frequency (how often) of vibrissal and eyelid movements is important. However, in the present study we focused on measuring the synkinesis of eye closure and whisking in different experimental sets. We think that the intensity of the synkinetic movement is more important factor. Analysis of synkinetic movements of the two main muscle groups of the face may provide valuable data on understanding recovery movements after facial nerve injury. Additionally we think that there is no need to setup such sophisticated systems, that is, laser system, to measure the synkinesis. We also proved that our video-based motion analysis system can be used to analyze synkinetic movements.

## 5. Conclusions

In our previous study [25], we found that HFA+Y-tube reduced collateral axonal branching. This reduction, however, did not result in improved whisking function (increased amplitude) nor in reduced polyinnervation of the levator labii superioris muscle [25]. We now extend these studies and show that reduced synkinesis of the orbicularis oculi and vibrissal muscles after HFA+Y-tube conduit correlates with the reduction in collateral axonal sprouting shown previously in the same animals [25].

In conclusion, the analysis of our video material revealed that reconstruction of the hypoglossal nerve with an injured facial nerve using a Y-tube conduit results in lower synkinetic activities of the orbicularis oculi and vibrissal muscles compared to when reconstruction is undertaken using simple end-to-end anastomosis. Surgical reconstruction that enables transected axons to regenerate in an unforced manner towards their targets via a Y-tube conduit may both reduce collateral axonal branching and synkinetic movement and improve function.
